# Candidate biomarkers from the integration of methylation and gene expression in discordant autistic sibling pairs

**DOI:** 10.1038/s41398-023-02407-4

**Published:** 2023-04-03

**Authors:** Samuel Perini, Michele Filosi, The Italian Autism Network, Giovanni Allibrio, Ilaria Basadonne, Arianna Benvenuto, Serafino Buono, Carmela Bravaccio, Carlo Casonato, Elisa Ceppi, Paolo Curatolo, Bernardo Dalla Bernardina, Lucio Da Ros, Francesca Darra, Anna Eusebi, Alessandra Gabellone, Andrea De Giacomo, Grazia Distefano, Federica Donno, Maurizio Elia, Elisa Fazzi, Michela Gatta, Stefania Giusto, Serenella Grittani, Evamaria Lanzarini, Giovanni Malerba, Elisa Mani, Barbara Manzi, Lucia Margari, Lucia Marzulli, Gabriele Masi, Paola Mattei, Luigi Mazzone, Massimo Molteni, Pierandrea Muglia, Sebastiano Musumeci, Antonio Narzisi, Antonio Pascotto, Cinzia Pari, Antonia Parmeggiani, Maria Giuseppina Petruzzelli, Alessia Raffagnato, Emiliangelo Ratti, Maria Paola Rossaro, Maria Pia Riccio, Paolo Rizzini, Renato Scifo, Martina Siracusano, Raffaella Tancredi, Alessandra Tiberti, Elisabetta Trabetti, Annalisa Traverso, Paola Venuti, Leonardo Zoccante, Alessandro Zuddas, Enrico Domenici

**Affiliations:** 1 Department of Cellular, Computational and Integrative Biology (CIBIO), University of Trento, Trento (TN), Italy; 2 EURAC Research, Bolzano, Italy; 3 Fondazione The Microsoft Research - University of Trento Centre for Computational and Systems Biology (COSBI), Rovereto (TN), Italy; 4 Unit of Child Neurology and Psychiatry, ASST Spedali Civili of Brescia, Brescia, Italy; 5 Department of Psychology and Cognitive Sciences, University of Trento, Rovereto, Italy; 6 Child Neurology and Psychiatry Unit, Tor Vergata University of Rome, Rome, Italy; 7 Oasi Research Institute - IRCCS, Troina, Italy; 8 Department of Translational Medical Sciences, Federico II University, Naples, Italy; 9 Faculty of Law, University of Trento, Trento, Italy; 10 Child Psychopathology Unit, Scientific Institute, IRCCS Eugenio Medea, Bosisio Parini, LC Italy; 11 Child Neuropsychiatry, Department of Surgical Sciences, Dentistry, Gynecology and Pediatrics, University of Verona, Verona, Italy; 12 Fondazione Smith Kline, Verona, Italy; 13 Child Neuropsychiatry Complex Operative Unit, University of Bari “Aldo Moro”, Bari, Italy; 14 Department of Basic Medical Sciences, Neuroscience and Sense Organs, University of Bari Aldo Moro, Bari, Italy; 15 Center for Autism Spectrum Disorders, Child Psychiatry Unit, Provincial Health Service of Catania (ASP CT), Catania, Italy; 16 Child and Adolescent Psychiatry Unit, Department of Biomedical Sciences, University of Cagliari, Cagliari, Italy; 17 Department of Clinical and Experimental Sciences, University of Brescia, Brescia, Italy; 18 Children and Adolescents Neuropsychiatry Unit, Woman and Child’s Health Department, Padova University Hospital, Padova, Italy; 19 Center for Autism Spectrum Disorder - Child and Adolescent Neuropsychiatry Unit, Rimini - Romagna Health Department, Rimini, Italy; 20 Child and Adolescent Neuropsychiatry Unit, Infermi Hospital, Rimini, Italy; 21 Department of Neurosciences, Biomedicine and Movement Sciences, Section of Biology and Genetics, University of Verona, Verona, Italy; 22 IRCCS Stella Maris Foundation, Pisa, Italy; 23 GRIN Therapeutics Inc. New York, New York, USA; 24 Department of Mental and Physical Health and Preventive Medicine, University of Campania, Naples, Italy; 25 Child Neurology and Psychiatry Unit, IRCCS ISNB, S. Orsola-Malpighi Hospital, Department of Medical and Surgical Sciences, University of Bologna, Bologna, Italy; 26 Centro Ricerche Cliniche di Verona, Integrated University Hospital Verona, Verona, Italy; 27 Child and Adolescent Neuropsychiatry Unit, Maternal-Child Integrated Care Department, Integrated University Hospital Verona, Verona, Italy

**Keywords:** Autism spectrum disorders, Genomics

## Abstract

While the genetics of autism spectrum disorders (ASD) has been intensively studied, resulting in the identification of over 100 putative risk genes, the epigenetics of ASD has received less attention, and results have been inconsistent across studies. We aimed to investigate the contribution of DNA methylation (DNAm) to the risk of ASD and identify candidate biomarkers arising from the interaction of epigenetic mechanisms with genotype, gene expression, and cellular proportions. We performed DNAm differential analysis using whole blood samples from 75 discordant sibling pairs of the Italian Autism Network collection and estimated their cellular composition. We studied the correlation between DNAm and gene expression accounting for the potential effects of different genotypes on DNAm. We showed that the proportion of NK cells was significantly reduced in ASD siblings suggesting an imbalance in their immune system. We identified differentially methylated regions (DMRs) involved in neurogenesis and synaptic organization. Among candidate loci for ASD, we detected a DMR mapping to *CLEC11A* (neighboring *SHANK1*) where DNAm and gene expression were significantly and negatively correlated, independently from genotype effects. As reported in previous studies, we confirmed the involvement of immune functions in the pathophysiology of ASD. Notwithstanding the complexity of the disorder, suitable biomarkers such as *CLEC11A* and its neighbor *SHANK1* can be discovered using integrative analyses even with peripheral tissues.

## Introduction

Autism spectrum disorders (ASD) are diagnosed in children at a young age if they exhibit difficulties in social interaction and communication and have restricted interests and repetitive behaviors [1, 2]. Affected children are ~1–2% of the population, and the prevalence is at least three times higher in boys than in girls [3]. Genetic studies have been instrumental in the discovery of multiple types of risk variants and the gain of new insights into the biology of ASD [4–7]. It has been acknowledged in several studies that ASD liability likely depends on additive effects of common and rare variants [8–11], and recent sequencing efforts have led to the identification of over 100 putative ASD risk genes, the majority of which are neuronally expressed [7]. Consistently, post-mortem investigations on brain samples from ASD subjects have revealed gene expression signatures associated with multiple synaptic functions [5, 12–14], in addition to a dysregulation of immune function genes, which has also been shown by investigations conducted in blood [15–19]. However, like for other complex disorders, genetic variants (including structural and chromosomal variations) do not fully explain the heritability of the disease, suggesting that the risk for the disease is not exclusively driven by genetic variants [20, 21].

DNA methylation (DNAm) represents one of the potential epigenetic mechanisms that may contribute to the risk of ASD due to interactions with genetic elements during the development of normal brain functions [22–26]. The interaction between ASD-associated genes and DNAm occurs through changes in the chromatin state driven by DNAm alterations that ultimately affect the expression of neurodevelopmental genes. As an example, Nguyen et al. provided strong evidence for epigenetic regulation via differential DNAm of the activity of two genes (*BCL-2* and *RORA*) in the autistic brain, validating their methylation status of *BCL-2* and *RORA* in lymphoblastoid cells of discordant monozygotic ASD twins and unaffected siblings [27]. Addressing the relationship between DNAm and expression of disease-associated genes is crucial for a full understanding of the etiology of heterogeneous diseases, but for ASD this relationship has been examined for a limited number of risk genes [28–31].

Genes may be up-or down-regulated as a response to modifications of DNAm that are linked to environmental variables and DNA sequence variations. Environmental variables such as advanced paternal age and chemical exposures have been shown to have strong effects on DNAm profile, and at the same time to increase the risk for ASD diagnosis [28, 32, 33]. Genetic variants may also influence DNAm levels through allele-specific methylation or methylation quantitative trait loci (mQTL), as it has been shown by large-scale investigations conducted in blood [34–37] and in brain [38, 39]. Of note, mQTLs have been found to be highly correlated between independent brain and blood samples [40], opening the possibility to learn about epigenetic mechanisms in the brain by studying peripheral tissues, and identifying biomarkers that reflect complex epigenetic interactions contributing to ASD risk, which would be otherwise overlooked.

Diagnosis of ASD still relies on the fulfillment of descriptive criteria, even though there is a strong motivation to develop effective biomarkers that can be used for early diagnosis. Some of the main candidate biomarkers for ASD have been identified through differential DNAm analysis. The receptor of the oxytocin hormone is one well-studied example. Its genomic region has been found differentially methylated in several studies with ASD and non-ASD controls, but the reported effects of DNAm on gene expression and protein synthesis have not been consistent across studies [41–43]. Differential DNA methylation analyses have been conducted in post-mortem tissues from ASD subjects and neurotypical controls, resulting in the identification of signatures of DNAm alterations and multiple biological pathways involved [44–46]. For biomarker discovery, brain tissues are less suitable than peripheral ones as they are much less accessible, and they also tend to be affected by post-mortem degradation. However, it must be taken into account that epigenetic signatures tend to be tissue-specific: interindividual variation in whole blood is not a strong predictor of interindividual variation in the brain, although DNA methylation in whole blood significantly co-varies with that in the brain at some genomic loci, including loci relevant for neurodevelopmental disorders [34, 47]. Therefore, whilst on the one hand, peripheral tissues might represent a valid alternative to brain tissues for detecting and developing effective biomarkers for ASD, on the other hand, they contain only a small amount of information that is shared with brain samples [48] and detecting significant signals may require very large sample sizes [49].

Here, we examined methylation data from whole blood samples of ASD discordant siblings that belonged to the Italian Autism Network (ITAN) cohort. We aimed to detect regions of the genome showing discordant levels of DNAm which correlated with the expression of nearby genes. As this association between diagnosis, DNAm, and gene expression is likely to be biased by demographic factors [50], genetic variants [34], biological and non-biological variables [46, 51, 52], we estimated the difference in DNAm between ASD and non-ASD siblings, and also the correlation between DNAm and gene expression conditionally on these confounding factors. Finally, we investigated biological pathways that were predicted to be significantly affected by the identified DNAm alterations.

## Subjects and methods

### Subjects

For the ITAN collection, thirteen centers were involved after approval by the Verona Hospital Ethical Review Board (study protocol AUT-SFK001, CE1419) and by the Ethical Review Committees. More than 800 individuals across 256 families were recruited and diagnosed with ASD with their consent or the parents’ consent [53]. The diagnosis was performed by experienced child psychiatrists that followed the DSM IV [54] and used standard tools: Broader Phenotype Autism Symptom Scale (BPASS), Autism Diagnostic Interview-Revised (ADI-R), Autism Diagnostic Observation Schedule (ADOS), and Krug Asperger Disorder Index screening. Of the ITAN collection, we selected 76 families having two siblings discordant for ASD diagnosis, and the affected child between 4 and 18 years old. The total number of subjects was 152 (Supplementary Table 1).

### DNAm profiling

DNA was extracted from peripheral blood samples using the Puregene Blood Kit (Gentra Systems, Minneapolis, MN, US), a modified salting-out precipitation method, following the manufacturer’s instructions. Each DNA sample was then checked for quality and quantity using NanoDrop ND 1000 spectrophotometer (Thermo Scientific, Wilmington, DE, US). Following sample randomization, DNA methylation quantification was performed at Life & Brain GmbH, Bonn, in two main steps. Firstly, DNA samples were submitted to bisulfite conversion using the EZ-96 DNA Methylation-Lightning^TM^ MagPrep protocol (Zymo Research, California, USA). This protocol consisted of adding the ready-to-use Lightning Conversion Reagent directly to the DNA coupled to a magnetic bead-based clean-up method. Secondly, the methylation profile of the converted DNA was determined across the human genome (hg19) using the Infinium HumanMethylationEPIC (“EPIC”) array by Illumina [55]. The DNA methylation data that support the findings of this study are available from the ITAN Foundation (see https://www.fondazioneitan.org/en for details) upon submission of an official request. Data are released only for research purposes, upon assessment of a project proposal by the ITAN Scientific Committee.

### DNA array genotyping and transcriptome analysis

DNA samples were genotyped by the Autism Sequencing Consortium [56] using Illumina GSA v2 arrays (~658,000 markers). Genotype data were called using the genotyping command line interface of Illumina array analysis platform. Transcriptome data were generated by Poly-A RNA sequencing on Illumina RNASeq Platform as previously described [17]. Exome sequence data were also available as a result of a collaboration with the Autism Sequencing Consortium [57].

### EPIC array data processing

The analysis of the EPIC array data was developed from the pipeline described in the ChAMP R package [58]. Raw IDAT files were imported and checked for quality using the minfi R package [59]. The quality control steps included default filters for probes with a detection *P*-value > 0.01 in at least one sample, for probes with a bead count < 3 in at least 5% of samples, for probes that were not found in CpG islands, for SNP-related probes according to the general recommendations [60], for cross-reactive probes [61, 62], for probes that aligned to multiple locations and finally, for probes located in chromosome X and Y. For each probe that passed the quality filters (i.e., CpG site), we calculated the Beta-value for each sample by taking the ratio between the number of methylated cytosines and the total number of cytosines at the specific CpG site. We then run BMIQ intra-array normalization [63], which is an effective method for adjusting Beta-values for bias introduced by the Infinium type 2 probe design [64]. We visualized the similarity of samples based on the normalized Beta-values of the 1,000 most variable probes using a multidimensional scaling plot, and we removed outlier families from downstream analyses if the metadata were incorrectly reported.

The contribution of batch effects to DNAm variation was determined using singular value decomposition (SVD [65]), and we used ComBat [66] to correct the Beta-values for technical features that showed a significant association (*P*-value < 0.05) with the first 20 principal components of DNAm variation. These batch effects were slide, plate, and array number, and we ensured that the biological variable of interest (ASD vs. non-ASD) was not confounded with these technical variables [67–69]. We also applied control probe adjustment [70] to further reduce the effects of technical biases on the methylation signal. This specific step involved the use of signal intensities for the EPIC array control probes which were representative of the efficiency of bisulfite conversion, and other aspects related to the chemistry of methylation quantification. We performed a principal component analysis in R [71] of these control probe intensities to deal with the high correlation between them, and then we determined whether there were significant batch effects of these principal components using SVD.

### Peripheral cell-type composition

We calculated cell-type composition for each sample from the Beta-values using the RefbaseEWAS method implemented in ChAMP [72]. We first assessed whether there was a significant difference in cell-type composition between ASD and non-ASD subjects using Dirichlet regression in R. Then, we extracted the variation in cell-type composition that was not partly explained by the diagnosis (i.e. the residuals of the Dirichlet regression), and we used these residuals of cell-type composition as covariates of a linear model to examine changes in DNAm that were not driven by cell-type heterogeneity (see below).

### Differential methylation

After filtering probes and samples for quality and correcting Beta-values for batch effects and cell-type composition, the next step of the DNAm pipeline was to identify differentially methylated probes (DMPs) using the limma R package [73, 74] implemented in ChAMP. For each probe, a linear model was fitted to the distribution of Beta-values across samples, with age, sex, family, and diagnosis as covariates. By including family in the linear model, we also expected to capture most of the variation explained by ethnicity because, for most samples, ethnicity was the same within families and different between families. A moderated gene-wise variance was then computed based on the empirical Bayes method [74] to test the null hypothesis that a difference in fitted values between ASD and non-ASD subjects was equal to zero. We called a CpG site a DMP if such a difference had a Benjamini-Hochberg (BH) corrected *P*-value < 0.05.

To address the interactions between neighboring CpG sites and the net effect that these sites have on associated gene(s), we determined the methylation status of clusters of probes by running the DMRcate algorithm [75] built in ChAMP. This algorithm identified differentially methylated regions (DMRs) between ASD and non-ASD subjects by applying a Gaussian smoothing to the moderated statistics of the limma output using windows of 1000 bp (default setting). DMRcate does not require a priori annotations of the genome for calling DMRs, and it does not combine genomically nearby CpG sites based on the direction of DNAm. Because of these two features of the algorithm, we were able firstly to study genomic regions that either had (e.g., promoter) or did not have (e.g., intergenic) explicit gene associations and, secondly, to analyze regulatory regions that showed hypermethylated and hypomethylated probes in ASD cases compared to non-ASD siblings (or vice versa). We used a quantile-quantile (Q–Q) plot between observed DMP/DMR *P*-values and expected *P* values under a uniform distribution of range [0,1] to check for spurious estimated *P*-values.

To assess the accuracy of the identified DMRs to classify ASD subjects, we first built a classifier model based on a Random Forest (RF) algorithm using the R package randomForest [76] and fed it with the median DNAm values of the DMRs. The model was trained and tested on 70% of the samples using a 5-fold cross-validation that was repeated ten times (R package caret [77]). The remaining 30% of the samples were used for validating the model classifier performance to predict ASD diagnosis. To avoid leakage of information from the test set into the training set, we then run differential methylation analysis on 70% of the samples, and then tested the ability of the identified DMRs to correctly classify the remaining 30% of the samples in terms of ASD diagnosis. The classifier was based on a Random Forest (RF) algorithm using the R package randomForest, and fed with the median DNAm values of the top 50 DMRs. A 5-cross-validation procedure was repeated ten times as above.

### Gene set enrichment

Both DMP and DMR datasets were analyzed for enriched gene sets to find biological functions significantly affected by differential DNAm between ASD and non-ASD subjects. We performed gene set enrichment analysis (GSEA) using bioinformatic tools that were developed explicitly for either DMPs or DMRs and that allowed us to correct gene sets *P*-values for the different numbers of CpGs between genes (i.e., probe number bias [78]).

For DMPs, GSEA was performed using functional class scoring, which is an approach implemented in the methylGSA R package [79]. Compared to other GSEA methods such as over-representation analysis, functional class scoring allowed us to rank all CpGs and not only DMPs by their *P*-values and, thus, to use the entire list of CpG-associated genes for each gene set to determine which biological functions were significantly influenced by differential DNAm between ASD and non-ASD subjects. Gene sets and the corresponding biological functions were extracted from widely used databases (Gene Ontology, KEGG, and Reactome) and also from specialized databases which were composed of genes implicated in psychiatric disorders (Gandal et al. [80]. and SFARI Gene database [81]), genes highly expressed in the human brain (The Human Protein Atlas [82]) and finally, genes involved in synapse functions (SynGO [83]).

For DMRs, we used an empirical Bayes GSEA method implemented in ChAMP [84] that has been developed to overcome the probe number bias and to avoid using only CpGs below an arbitrary significance threshold [85]. All CpGs of each DMR were mapped to genes, and these genes were ranked by their overall level of differential DNAm. This list of ranked genes was then examined for enriched biological pathways which were obtained from widely used databases (Gene Ontology, KEGG, and Reactome).

### Integration with gene expression and genotype data

To examine the potential effects that differential DNAm may have on transcription activity and gene regulation, we integrated the DNAm profile with gene expression data from the same set of samples [17] using the ELMER R package (version 2.8.3) [86]. This type of analysis was performed in supervised mode. From the list of DMPs generated using limma (see above), distal EPIC probes (at least 2 Kb far from transcriptional start sites) were kept for the integration with gene expression data if the DNAm difference at these probes was |0.03| between ASD and non-ASD subjects. Each differentially methylated distal probe was then paired with the closest ten upstream genes and the closest ten downstream genes. For each probe-gene pair, we computed the inverse correlation between the DNAm of the probe, and the expression of the gene and further analyzed the difference in gene expression between ASD and non-ASD subjects using the Mann-Whitney U test. We used the Benjamini-Hochberg corrected *P*-values for detecting probe-gene pairs that may be potentially associated with ASD diagnosis.

The correlation between DNAm and gene expression may also depend on genetic components. To understand potential genetic effects on DNAm and gene expression, we used the genotype data of our subjects and integrated them with published data of independent DNAm quantitative trait locus (mQTL) [34] and of *cis*-expression quantitative trait loci (eQTL) [87]. First, we searched for *cis*-eQTL of the gene which was significantly associated with the differentially methylated distal probe. Then, we checked if such a genetic variant was also a mQTL. If a given SNP was a mQTL and corresponded to our distal probe, we estimated its effect on DNAm using a beta regression model [88] where the independent variables were the diagnosis and the genotype of the subjects multiplied by the Beta-value of the mQTL. Each linear model included two different top-ranked mQTL which were estimated to be in linkage equilibrium using LDlink [89].

### Correlation between methylation in blood and in reference brain datasets

We obtained data from the Image-CpG database [90] to assess to which extent the methylation level within the identified DMRs could reflect brain methylation. Blood-brain correlations parameters for EPIC Illumina EPIC array were available as summary statistics. As described in Roberson-Nay et al. [91] we computed the median rho correlation, together with the minimum and maximum rho, for all CpGs included in each DMR.

## Results

### Difference in cell-type composition

**Fig. 1:**
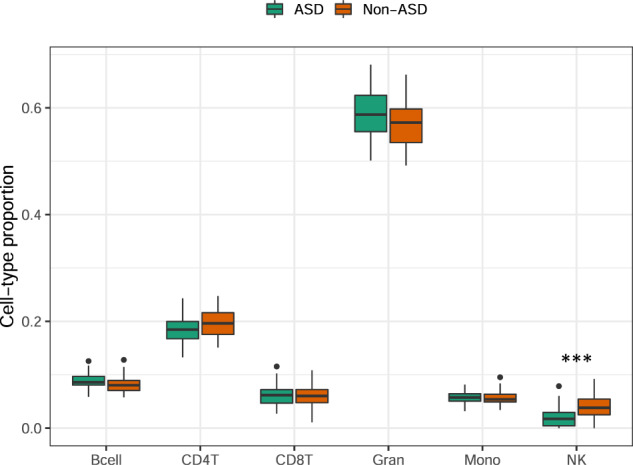
Estimated cell-type proportions in ASD and non-ASD subjects. Statistical significance was calculated using Dirichlet regression and only the proportions of NK cells were significantly different between ASD (green) and non-ASD (orange) subjects (*** meaning a *P*-value < 0.001). The error bars represent the standard deviation.

We estimated per-sample cell-type proportions from DNAm data and analyzed these compositional data using Dirichlet regression to determine whether there was a difference between ASD and non-ASD subjects. We found no significant difference in estimated cell-type composition, except for NK cells where the proportion was significantly lower in ASD subjects compared to non-ASD subjects (ASD = 0.01, non-ASD = 0.04, *P*-value < 0.001) (Fig. 1; Supplementary Table 2). These differences in cell-type composition were consistent with the results of a previous study on ASD in which the same subjects were analyzed using gene expression data [17]. Even though we performed a DNAm-based cell-type deconvolution method, our estimated proportions were correlated (Pearson’s *r* > 0.25) with the proportions that were calculated in the previous study where it was used an RNA-based deconvolution algorithm (Supplementary Fig. 1a, b).

### Identification of DMPs and DMRs

We examined EPIC methylation arrays of 76 discordant autistic sibling pairs to understand the role of epigenetics in ASD. We identified two outlier families using a multidimensional scaling plot based on DNAm Beta-values (Supplementary Fig. 2), but we discarded only one outlier family in which the sampling was not performed on the same date for both siblings; in all the other families, each pair was sampled on the same date. After removing this outlier, the methylation profile of the 75 families was overall homogeneous, and largely shared between discordant siblings of the same family (Supplementary Fig. 3). Notably, one subject is carrier of a 15q11.2-13.1 duplication and three ASD subjects carry a de novo protein truncating mutation in one of the 72 associated genes identified by Fu et al. [57]. However, since all of them clustered with the corresponding control sibling in the multivariate analysis (Supplementary Figs. 2, 3), we retained them in the analysis. Family was, in fact, one of the factors that resulted to be significantly associated with the first 20 principal components of the SVD analysis. The other factors that explained a significant variation in Beta-values were either involved with the array processing (e.g., slide and control probes intensities) or with the heterogeneity of cell types (e.g., granulocytes and B cells), and no significant variation was explained by the diagnosis (Supplementary Fig. 4). However, after fitting a linear model to each probe’s Beta-values to correct for family, age, sex, batch effects, control probes intensities, and cell-type composition, we were able to capture the portion of DNAm variation that was significantly associated with the diagnosis (Supplementary Fig. 4).

**Fig. 2:**
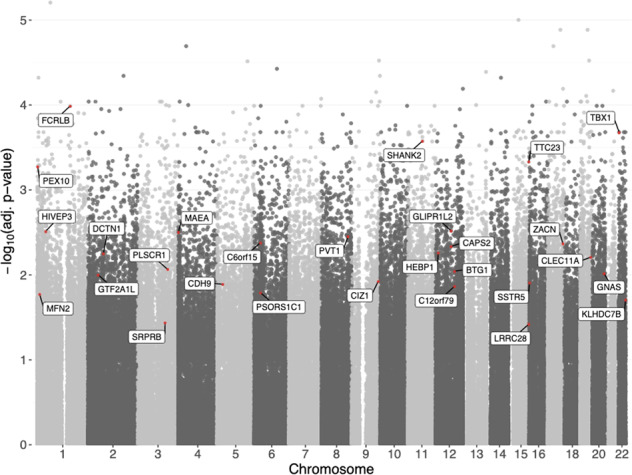
Statistical significance of genome-wide differential methylation between ASD and non-ASD subjects. Top-ranked DMP (red point) of each top 2% DMR and its corresponding gene (text box).

For the differential methylation analysis between ASD and non-ASD siblings, we used the adjusted Beta-values and we identified 37,643 DMPs across the genome with a BH corrected *P*-value < 0.05 and a range of Beta-values difference between −0.05 and 0.05 (Supplementary File 1; for the Q–Q plot, see Supplementary Fig. 5). The data on single DMPs may not accurately reflect the interactions between neighboring CpG sites and the net effect that these sites have on associated gene(s). To overcome these limitations, we ran the DMRcate algorithm [75] and identified 418 DMRs with Stouffer’s *P*-value < 0.05 that were located genome-wide, as expected from the distribution of DMPs (Supplementary File 2). Of these DMPs and DMRs, the top-ranked sites were determined to potentially affect genes that have been reported to be involved in neurodevelopmental diseases such as *TBX1* [92], *SHANK2* [93], and *TTC23* [94] (see Fig. 2 and Supplementary Fig 6 which reports the location of the DMRs relative to the genes discussed here). Notably, out of the 418 significant DMRs, only 26 has a statistically significant difference when comparing males vs males (Stouffer’s *P*-value < 0.05, see Supplementary File 2), and none of the neurodevelopmental related genes here discussed displays a significantly different methylation in males vs females. To further address the potential confounding effect of sex on diagnosis-related findings, we additionally conducted an analysis stratified by sex (i.e. a separate analysis for males and females), see Supplementary File 3. Consistently with the smaller sample size of females compared to males, we detected fewer DMRs reaching statistical significance and larger *p*-values in females (641 DMRs in females and 7570 DMRs in males; 170 DMRs were shared between females and males). However, we found a strong correlation (Pearson’s correlation 0.96, *P*-value < 0.001) between male and female coefficients estimated by sex-stratified DMP analysis, see Supplementary Fig 7.

To assess the accuracy of the identified DMRs to classify ASD subjects, we then built classifier models based on a Random Forest algorithm as described in Methods. The first model was fed with the median DNAm values of the 418 DMRs identified by differential methylation on the full cohort. Despite the small differences in methylation between ASD and non-ASD siblings (see the mean values of DNAm for each DMR in ASD and non-ASD subjects and their distribution in Supplementary File 4) and the potential *p*-value inflation, the overall model performance was 0.758 in terms of AUC. Since the performance of the first model can be skewed away from the true performance due to leakage of information from the test set into the training set, we performed a second model based on DMRs differential methylation analysis on 70% of the samples, and then tested the ability of the identified top 50 DMRs to correctly classify the remaining 30% of the samples in terms of ASD diagnosis, as described in Methods. In this case, the model performance was 0.72 (see Supplementary Fig 8). Among the newly identified DMRs, 42 had exactly the same genomic position of the DMR identified with the full cohort analysis. As expected, their mean difference between ASD and control subjects correlated with their feature importance in the RF-based classification (see Supplementary Fig 9). By restricting the classifier to these 42 DMRs (and feeding the classifier with their median DNAm values), the overall performance raised up to 0.75.

### Enriched biological processes in ASD

**Fig. 3:**
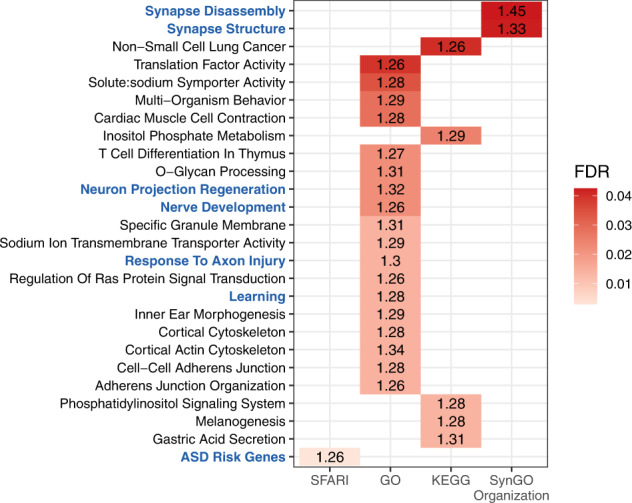
Top 1% enriched gene sets. Biological functions (*y*-axis) were extracted from four databases (*x*-axis) and they were ranked by FDR (red gradient) and Normalized Enrichment Score (values inside the tiles). Of the top 1% functions, seven may have an important contribution to the etiology of ASD (highlighted in blue).

We performed GSEA to understand which biological processes may have been altered by differential methylation between ASD and non-ASD subjects. We extracted biological processes from a total of seven databases (Gene Ontology, KEGG, Reactome, Gandal et al. [80], SFARI Gene [81], The Human Protein Atlas [82], and SynGO [83]), and identified 457 and 2,305 processes that were significantly represented (FDR < 0.05) by the list of genes associated with DMPs and DMRs, respectively (Supplementary File 5; Supplementary File 6). Of the top 1% significantly enriched processes with the highest Normalized Enrichment Score, two processes involved genes associated with synapse disassembly and structure, three processes were associated with neurogenesis, and one process involved learning genes. Finally, we found a significant enrichment for SFARI ASD risk genes (Fig. 3). To further corroborate the evidence of the enrichment of neurodevelopmental processes, we built a 2 × 2 contingency table with the number of index DMPs and non-DMPs (at gene level) that are associated with the top ranked neurodevelopmental processes (displayed in blue in Fig. 3), and the number of DMPs and non-DMPs associated with other processes (see Supplementary Table 3. We then assessed whether DMPs were overrepresented for neurodevelopment-related processes using a hypergeometric distribution (one side Fisher’s exact test). The result confirms that neurodevelopment-related processes are indeed enriched within the DMPs (odds ratio = 2.96, *P*-value < 0.001).

### Correlation between DNAm and gene expression

Differential methylation between different biological conditions may contribute to phenotypic differences through changes in the underlying gene expression. Here, we investigated whether ASD and non-ASD siblings showed genes or genomic regions that differed in the joint pattern of DNAm and gene expression using ELMER analysis in supervised mode. We identified 346,224 probes on EPIC arrays that were at least 2 kb away from a transcriptional start site. Six of these distal probes were hypermethylated and ten were hypomethylated in ASD subjects with FDR < 0.05 and Beta-values difference > |0.03 | (Supplementary Fig. 10). We then identified putative target genes for these differentially methylated distal probes and found a significant association for three of the hypermethylated probe-gene pairs and four of the hypomethylated probe-gene pairs (Table 1). Notably, we identified significant associations for *CLEC11A* and *MOB1A*, genes known to be associated with immune and mental disorders [95, 96].

**Table 1:** Probe-gene pair association using ELMER.

Hypermethylated	Gene	Distance	FDR
cg17729891	C19orf48	193401	0.01
cg20315590	TSEN15	−1959694	0.01
cg17729891	CLEC11A	119026	0.04
cg10841563	GNAQ	−2633643	0.01
cg10841563	KIF27	3171593	0.01
cg10841563	HNRNPK	3302978	0.01
cg05890377	MOB1A	21940	0.03

Hypermethylated (left) and hypomethylated (right) distal probes in ASD subjects and putative target genes.

**Fig. 4:**
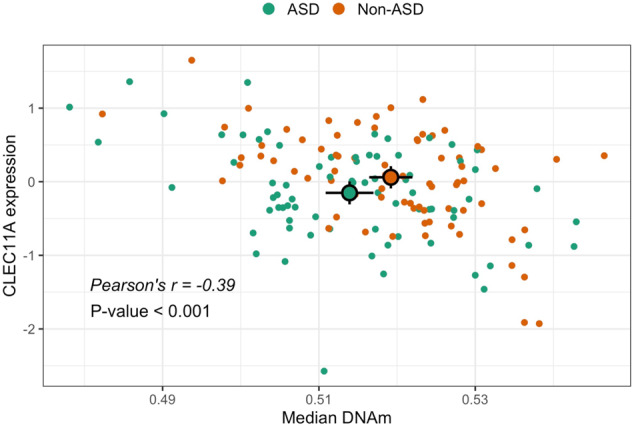
Gene expression and DNAm of CLEC11A. Gene expression (*y*-axis) was normalized and adjusted for batch effects and demographic parameters [17]. Median DNAm (i-axis) was calculated for the DMPs that were significantly associated with *CLEC11A*. Mean (large and black circles) and 95% confidence intervals (black bars) of CLEC11A expression and DNAm are shown for ASD (green; DNAm = 0.514 (0.511–0.517); DGE = −0.151 (−0.307–0.005)) and non-ASD (orange; DNAm = 0.519 (0.516–0.522); DGE = 0.061 (−0.091–0.212)) siblings.

Correlated DNAm and gene expression changes may be driven by genetic variants influencing DNAm at the nearby target gene. We identified two SNPs as candidate genetic variants for such an impact on DNAm of *CLEC11A* and *MOB1A*. We investigated the effects of the SNPs genotype on the estimated differential DNAm between ASD and non-ASD subjects, but only for *MOB1A* the genotype contribution was strong enough to remove the differential DNAm signal (Supplementary Table 4). For *CLEC11A*, we found that differential DNAm was not lost when taking into account the presence of a mQTL. This strong signal for *CLEC11A* was supported by both the DMR analysis in which the genomic region involving *CLEC11A* was characterized as DMR (Fig. 2; Supplementary File 2), and the estimated correlation between the median DNAm across the four DMPs at 200–1500 bases upstream of *CLEC11A* transcriptional start site and *CLEC11A* expression (Pearson’s *r* = −0.39, *P*-value < 0.001; Fig. 4). Although the difference in DNAm of *CLEC11A* between ASD and non-ASD siblings was significant, gene expression was only marginally different between the two groups (Fig. 4).

Finally, we exploited the dataset reported by Braun et al. [90] to investigate the degree of correlation between blood and brain for some of the identified DMRs, i.e. TTC23, TBX1, SHANK2 and CLEC11A, by computing the Spearman correlation for all CpGs included in each DMR. All four DMRs showed a trend for positive correlation between blood and brain methylation, with TTC23 displaying a consistent trend across all CpGs (see Supplementary Tab 5), suggesting the potential of using blood for detecting changes which may be relevant in the brain.

## Discussion

Identifying robust biomarkers for ASD has been a challenging task. This group of neurodevelopmental disorders is polygenic, phenotypically heterogeneous, and influenced by both genetic and environmental factors [97–100]. As there is no single common genetic variant showing a strong predictive value for ASD diagnosis [101, 102], candidate biomarkers have been searched for outside the protein-coding sequences of genes such as in enhancers and intergenic regions. In these regulatory regions, biomarkers can be found by detecting changes in DNA methylation (DNAm) that may potentially affect gene functions [22–26]. However, although the contribution of DNAm to the risk of ASD has been acknowledged, questions remain about the potential of this type of epigenetic biomarkers to help in the development of diagnostic and therapeutic approaches to ASD.

Here, we analyzed the methylation profile of whole blood samples from 75 sibling pairs with a discordant ASD diagnosis. We aimed to analyze DNAm in subjects with ASD and detect dysregulated genomic regions that may be involved in the etiology of the disorder. In addition, we investigated possible differences in cell-type proportions between ASD and non-ASD siblings that might have been involved in the pathophysiology of the disorder.

In our study, we analyzed the methylation profile of a subset of the ITAN collection, the same subset previously examined at the transcription level [17]. We estimated cell-type proportions using DNAm array data of whole blood mixtures and showed a reduced fraction of NK cells in ASD siblings. A similar reduction in NK cells was also found using transcriptome data of the same cohort [17], consistent with previous investigations reporting either a decrease of NK cells [103] or a reduction of their activity [104–107]. NK cells act against viral infections, representing an important defensive mechanism of the innate immune system [108], and their decreased level or activity would suggest a suboptimal function of the immune system in ASD. In ASD children, immune pathways are typically dysregulated, and the expression of genes that are involved in the immune response has been found to be correlated with changes of DNAm in their regulatory regions [109]. For example, Nardone et al. [46]. found that hypomethylated sites were associated with the overexpression of transcription factors regulating the development of the microglia. It has also been reported that the immune system of mothers of ASD cases responded slowly to infections due to DNAm alterations at regulatory regions of immune genes [110]. Such DNAm alterations may be inherited, potentially contributing to the risk of ASD in children of immunocompromised mothers. More recently, a genome-wide methylation analysis in the blood of ASD subject vs neurotypical controls has led to the identification of a putative epigenetically distinct subset of children driven by altered blood cell composition [111], further supporting the role of the immune systems in the pathophysiology of ASD. Nevertheless, given the known dynamic nature of the immune system and possible variations in cell proportions and subsets reflecting hormonal changes or environmental influences [112], the reduction in NK cells observed might well be reflecting a transient state, and would need to be confirmed in longitudinal studies.

Autism is a polygenic neurodevelopmental disorder and, besides immune genes, there are other genes that have been associated with ASD [5, 6, 8, 11, 19]. By comparing the genome-wide methylation profile of ASD subjects versus their healthy sibling controls, we identified hundreds of genes that were differentially methylated after accounting for demographic data, batch effects, and cell-type composition. In particular, 37,643 genomic sites (DMPs) and 418 regions (DMRs) were differentially methylated between ASD and non-ASD siblings. We detected DNAm alterations in *TBX1*, *SHANK2*, and *TTC23*, genes that have been previously shown to be affected by epigenetic processes in ASD and other neurodevelopmental diseases [92–94]. As expected, genes that have a significant contribution to the risk of ASD, such as *TBX1*, *SHANK2*, and *TTC23*, are regulated through DNAm, suggesting that the etiology of ASD can be better understood by also looking at DNAm alterations.

By conducting gene set enrichment analysis (GSEA), we showed that identified DMPs were associated with biological processes related to synapse functions and neurogenesis. Although neurodevelopmental pathways are preferably studied using brain tissues, our results support the potential for identifying epigenetic biomarkers of neurodevelopment even in peripheral tissues [47, 48].

Differences in cellular composition, DNAm, and function of neurodevelopmental pathways are expected to be associated with differences in gene expression between affected subjects and controls. Ultimately, the association between multiple mechanisms may strongly depend on genetic variants that, for example, are responsible for differential DNAm and the regulation of gene activity [113]. We integrated the methylation Beta-values of DMPs of our samples with the corresponding gene expression and genotype data to understand the potential relationship between genetic and non-genetic components in ASD. We first calculated the correlation between gene expression and DNAm, identifying a significant and negative correlation between DNAm and expression at the DMR that was mapped to *CLEC11A*. We then combined genotype data from the same samples and methylation eQTL data and showed that the correlation was not driven by genotype effects of candidate SNPs located in the same region.

In other studies, the region of *CLEC11A* has been identified as a candidate locus for the etiology of neurodevelopmental disorders due to its position nearby *SHANK1*, a gene strongly associated with ASD [96]. SHANK family genes encode postsynaptic proteins that are needed for functional electrochemical communication at the level of synapses, and mutations in any of the three genes (*SHANK1*, *SHANK2*, and *SHANK3*) have been reported to disrupt neuronal activity [96, 114]. Different SHANK mutations have been found in ASD individuals, including a deletion covering *CLEC11A* and *SHANK1* in males with ASD [96, 115, 116]. In addition, differential DNAm between ASD cases and controls has been determined at the regulatory regions of *SHANK1* and *SHANK3* [117, 118]. We could not detect DNAm changes that were specifically associated with *SHANK1* nor *SHANK3* because these biomarkers are absent in the blood, but we did find a DMR associated with the *SHANK2* gene which is instead also expressed in the blood [119], showing a trend for correlation with the methylation level assessed in brain datasets.

Compared to studies based on brain tissues, our analysis using whole blood samples was limited in its power to capture neurodevelopmental genes and pathways, as we could only examine those that were not exclusively regulated in the brain. Still, we found differentially methylated sites in the blood that were associated with genes involved in neurodevelopment and ASD. We are aware that for some of these candidate genes differential DNAm may reflect a correlation between ASD diagnosis and some other variables that were not measured in our study, and may be transient, thus further investigations would be required to validate our findings. Nevertheless, our results provide further support for the possibility of using peripheral tissues to identify candidate epigenetic ASD biomarkers through integrative analyses across genomic data, as in the case of the *CLEC11A*-*SHANK1* region. When validated, the methylation markers might become a valuable asset to support early diagnosis, particularly for non-syndromic or “idiopathic autism”, and assist the identification of epigenetically distinct subtypes of autism [111, 120], thus facilitating stratified therapies.

## Supplementary information

Supplementary Information

Supplementary File 1

Supplementary File 2

Supplementary File 3

Supplementary File 4

Supplementary File 5

Supplementary File 6
