# Supplementary material for: Candidate biomarkers from the integration of methylation and gene expression in discordant autistic sibling pairs
Source: Transl Psychiatry. 2023 Apr 3;13:109. doi: 10.1038/s41398-023-02407-4 (PMC10070641; doi:10.1038/s41398-023-02407-4)
Supplement: Supplementary file 1 — Supplementary Information [file 41398_2023_2407_MOESM1_ESM.docx]

**Perini et al. - Supplementary Information**
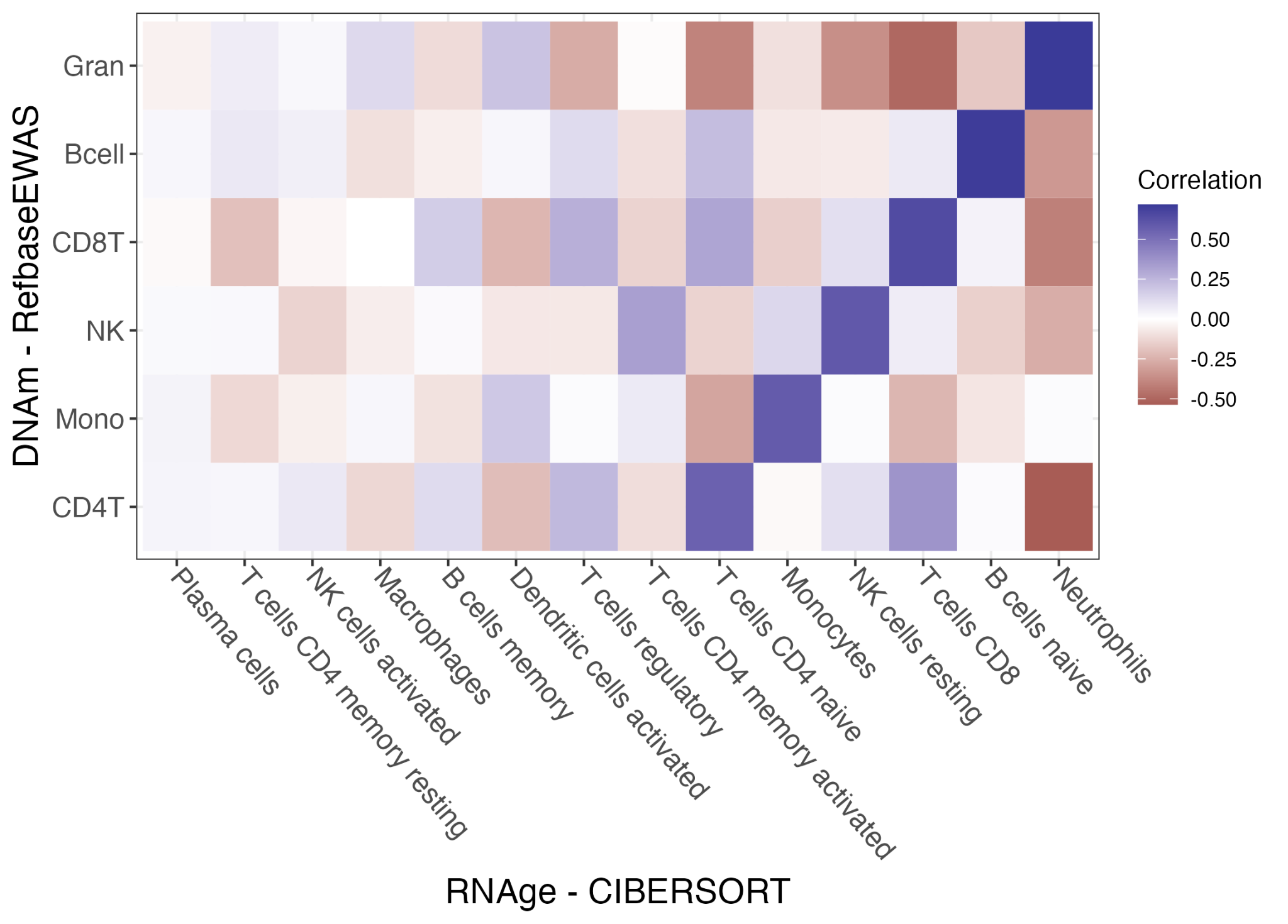


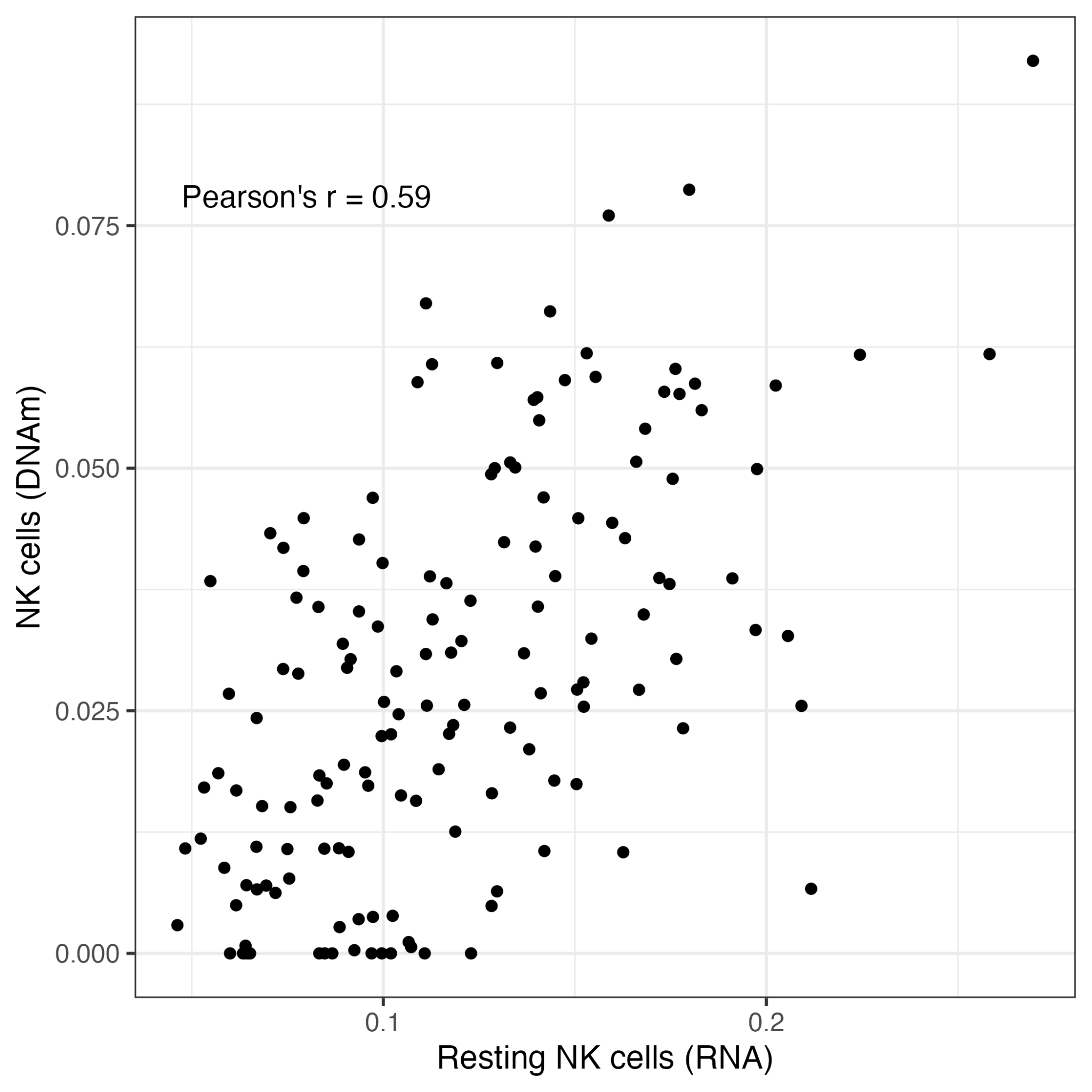


**Supplementary Figure 1 - Pearson’s correlations between two different cell-type deconvolution methods.** Houseman’s reference-based method was applied to DNAm (y axis) and the CIBERSORT output (x axis) was taken from a previous RNA Gene Expression study^1^. a) Top: correlation for all cell types; b) bottom: correlation for NK cells


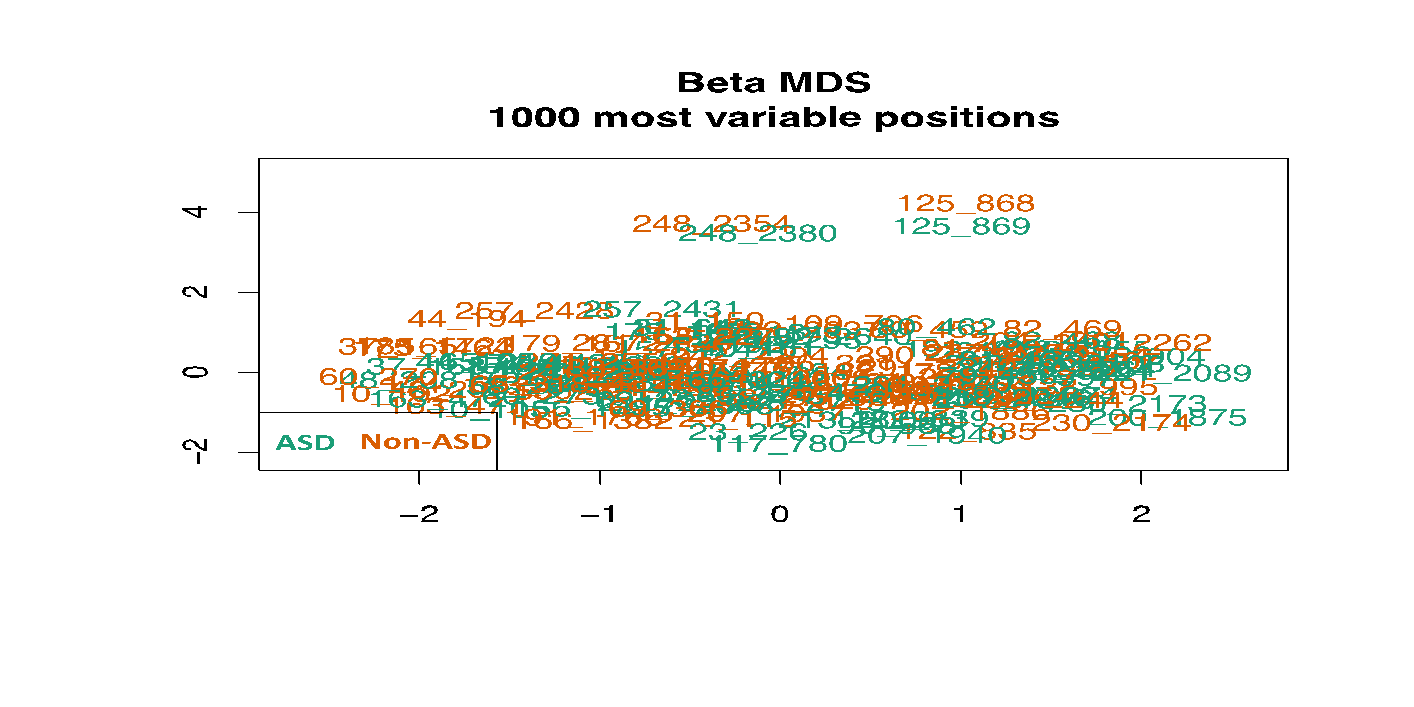
 **Supplementary Figure 2 - Multidimensional scaling plot.** Distribution of Beta-values of the 1,000 most variable probes before (left) and after (right) outlier removal. Sample IDs are colored by diagnosis (ASD in green and non-ASD in orange).
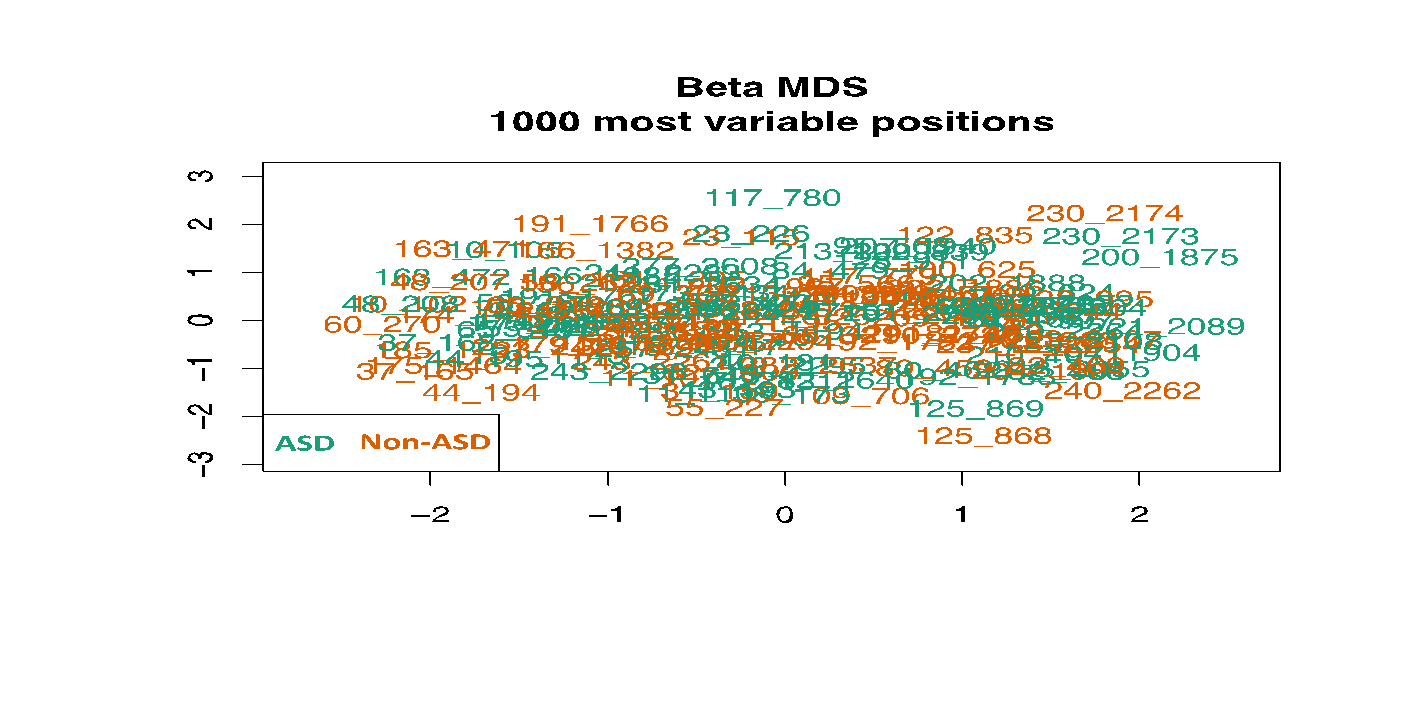


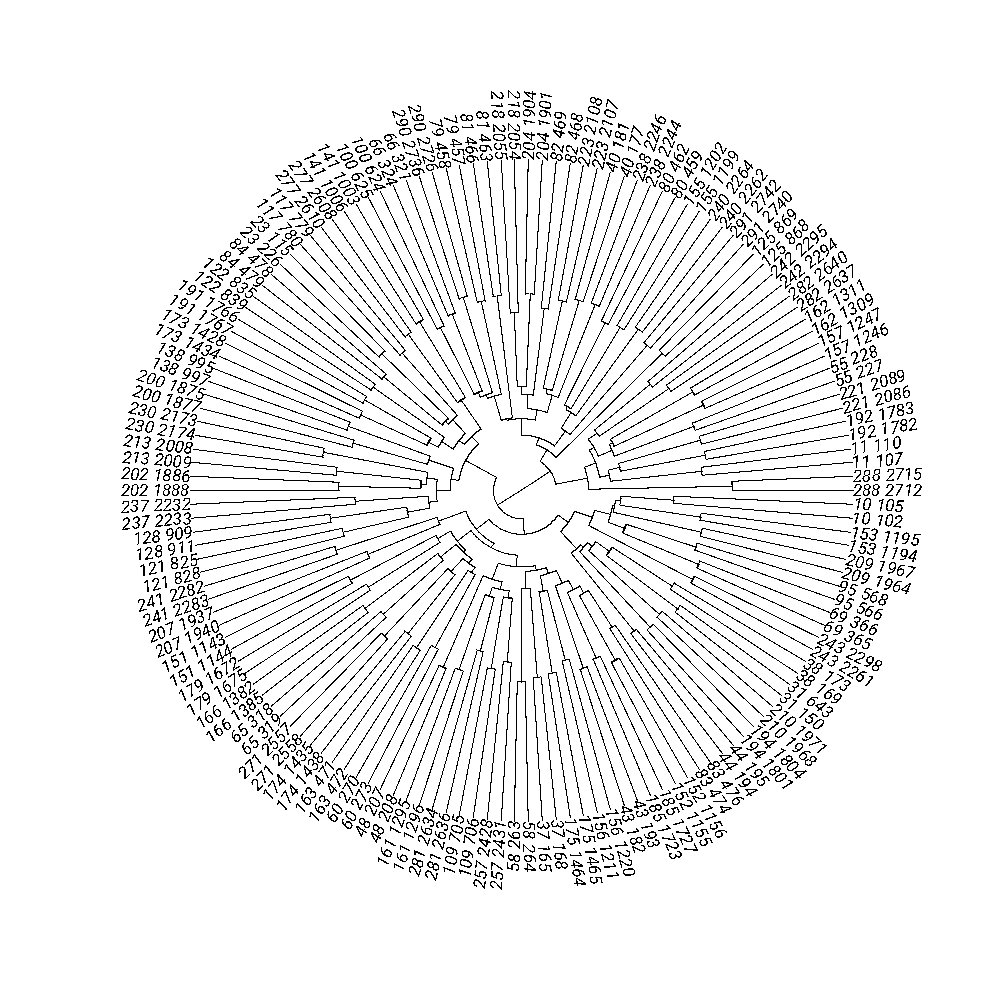


**Supplementary Figure 3** - **Dendrogram based on the Beta-values of the 1,000 most variable probes after outlier removal.** Siblings of the same family (i.e., sample IDs starting with the same number) are next to each other.


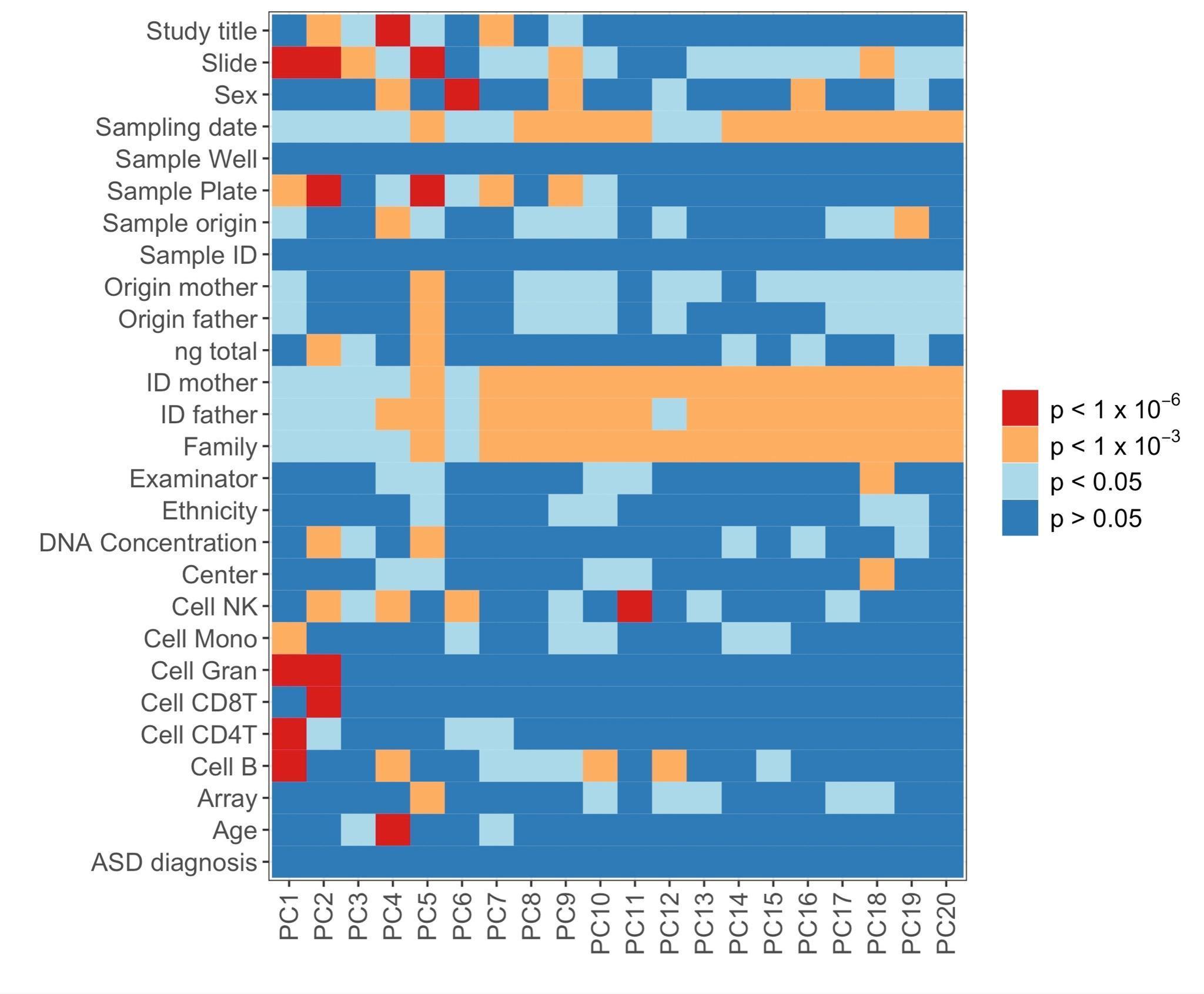

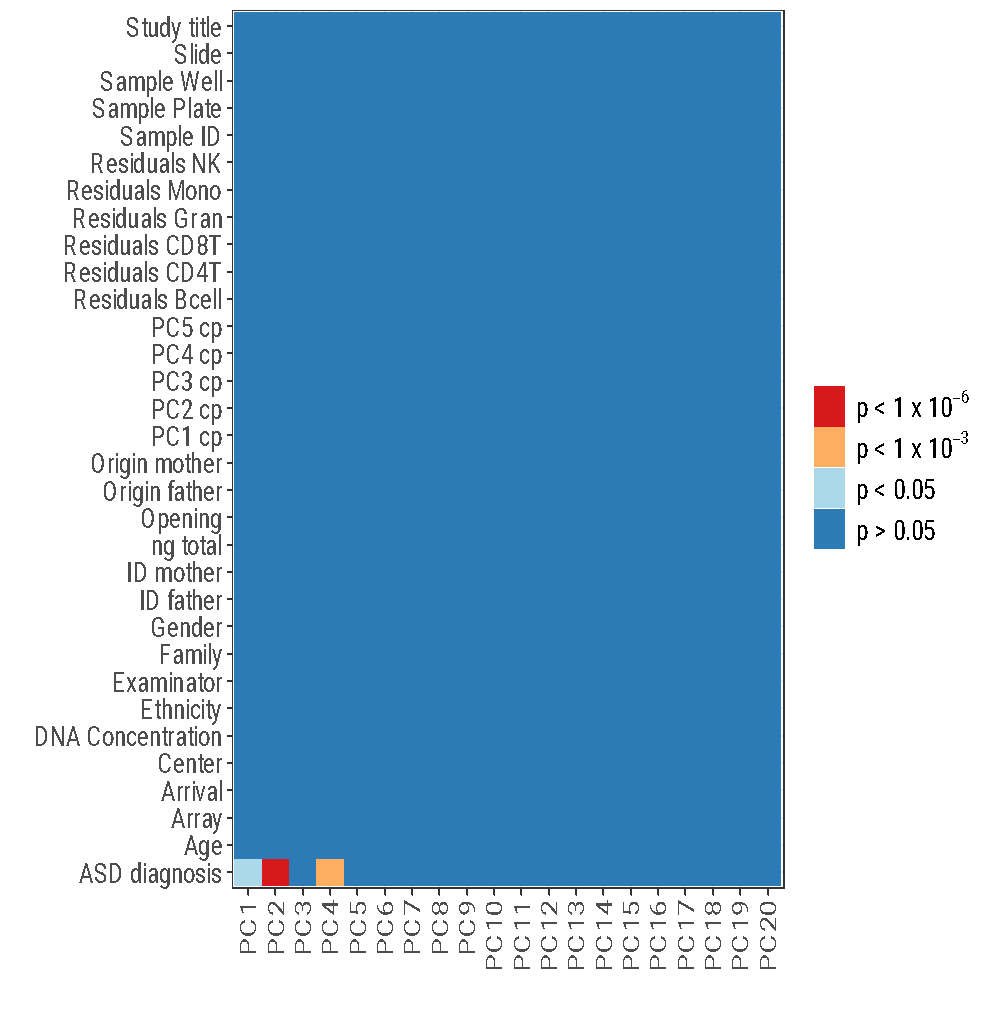


**Supplementary Figure 4 - Singular value decomposition of DNAm before (left) and after (right) variable correction.** Methylation Beta-values were adjusted for batch effects, the first five principal components of control probes intensities (“PCs cp”), cell heterogeneity (“Residuals cell-type”), age, gender and family (y axis). The associations between the first 20 principal components (x axis) and covariates (y axis) are color coded according to their statistical significance (from P-values > 0.05 in blue to P-values < 1 x 10^-6^ in red). Significance for categorical covariates was calculated using Kruskal test and for numeric covariates using linear regression.


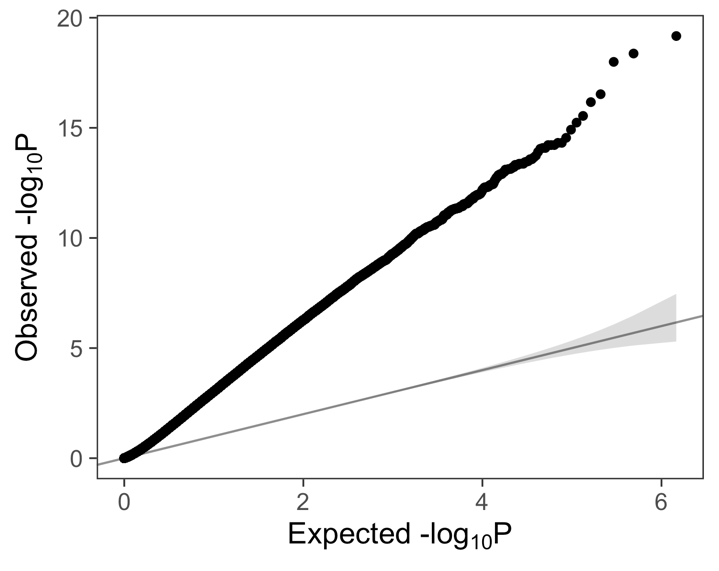

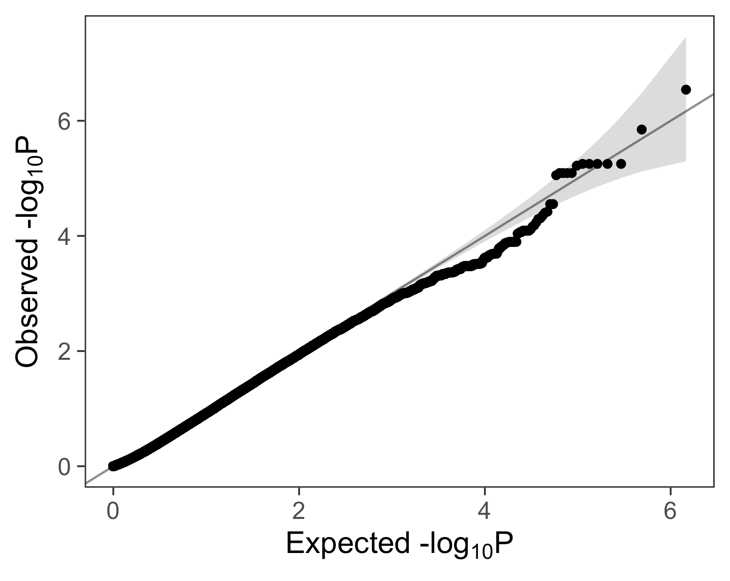


**Supplementary Figure 5 - Quantile-quantile (QQ) plot of DMPs’ adjusted P-values before (left) and after (right) variable correction.** The gray band represents the 95% confidence interval.

TTC23


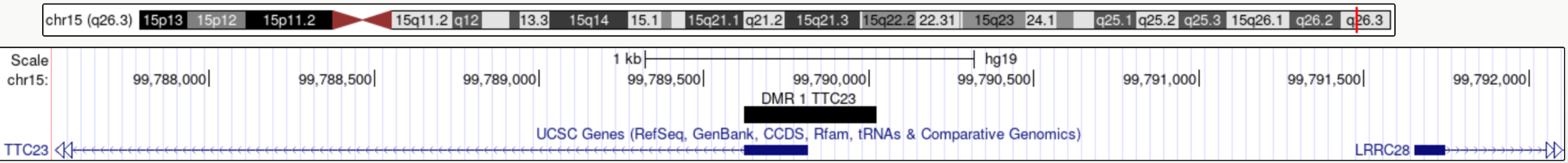


TBX1


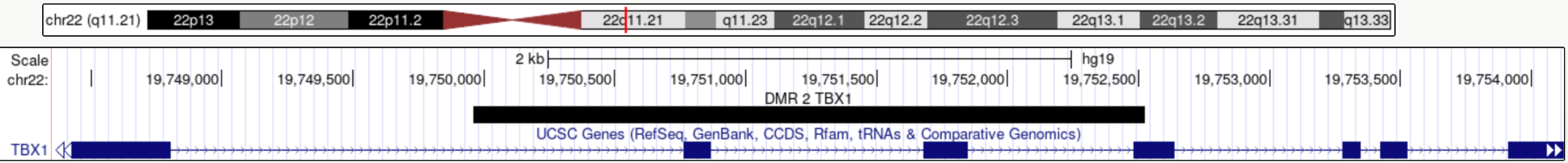


SHANK2


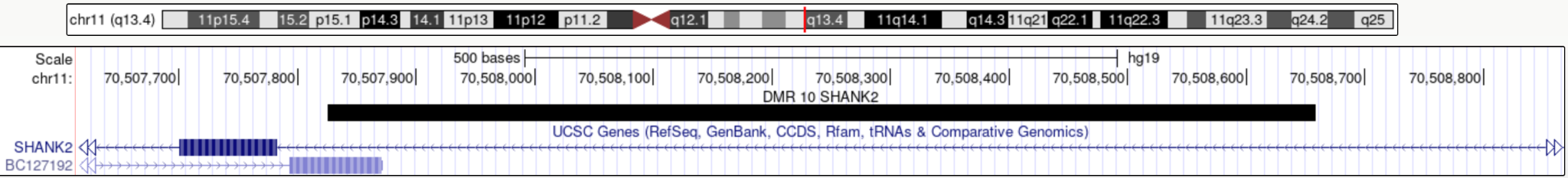


CLEC11A


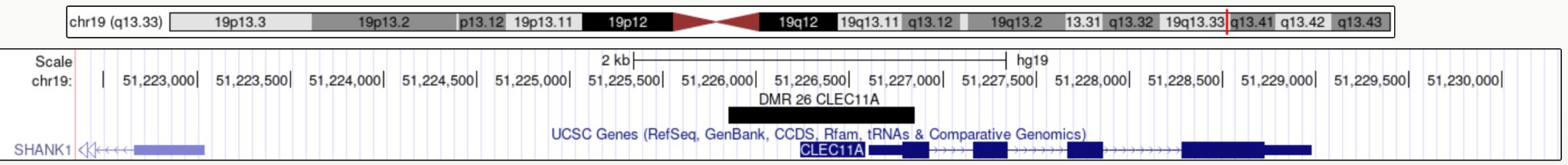


**Supplementary Figure 6 - Location of the DMRs relative to the key genes discussed in the text** (TTC23, TBX1, SHANK2 and CLEC11A)


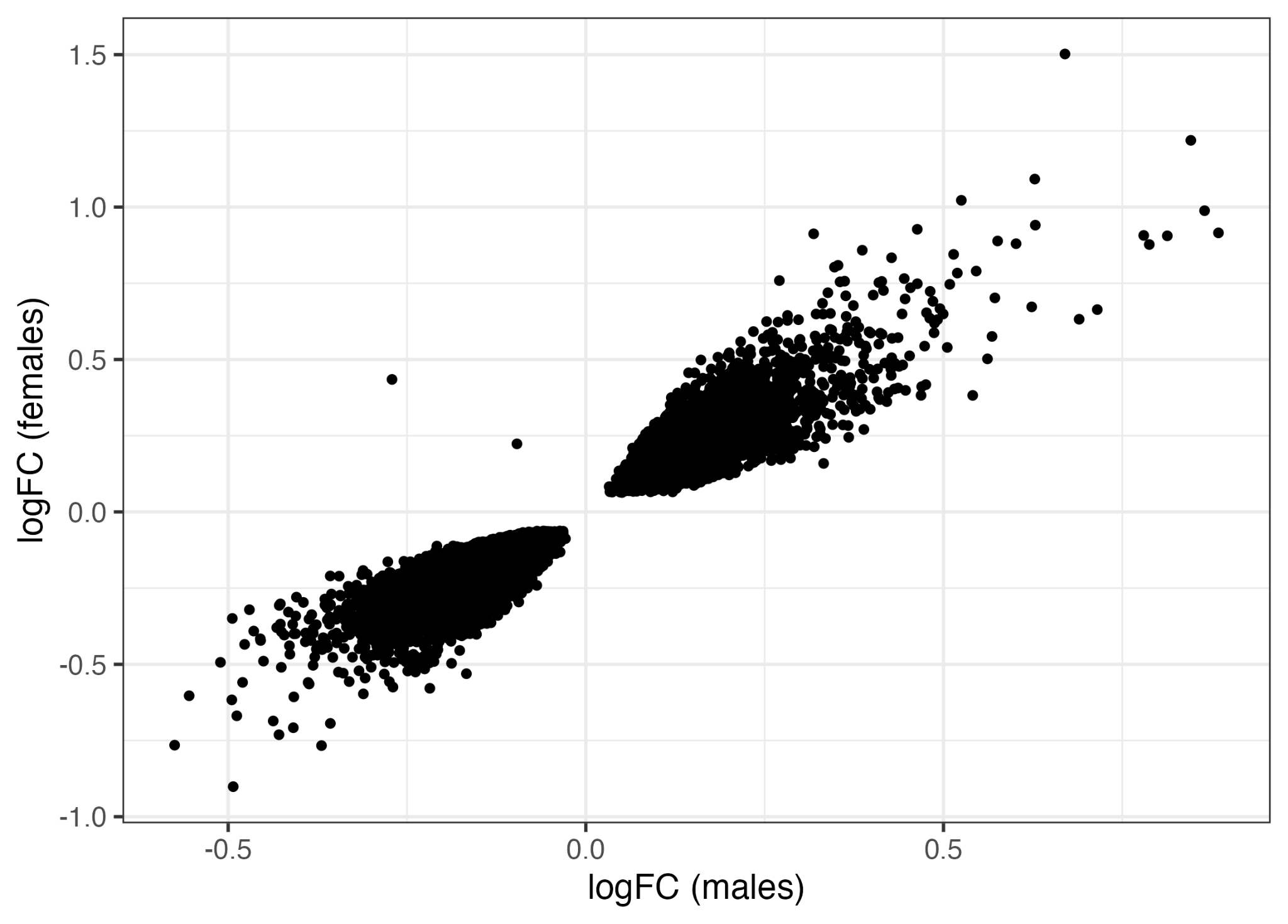


**Supplementary Figure 7 - Correlation between DMPs (ASD vs non-ASD subjects) identified in female vs male stratified analysis.** Pearson’s correlation between male and female coefficients was 0.96 (P-value < 0.001).


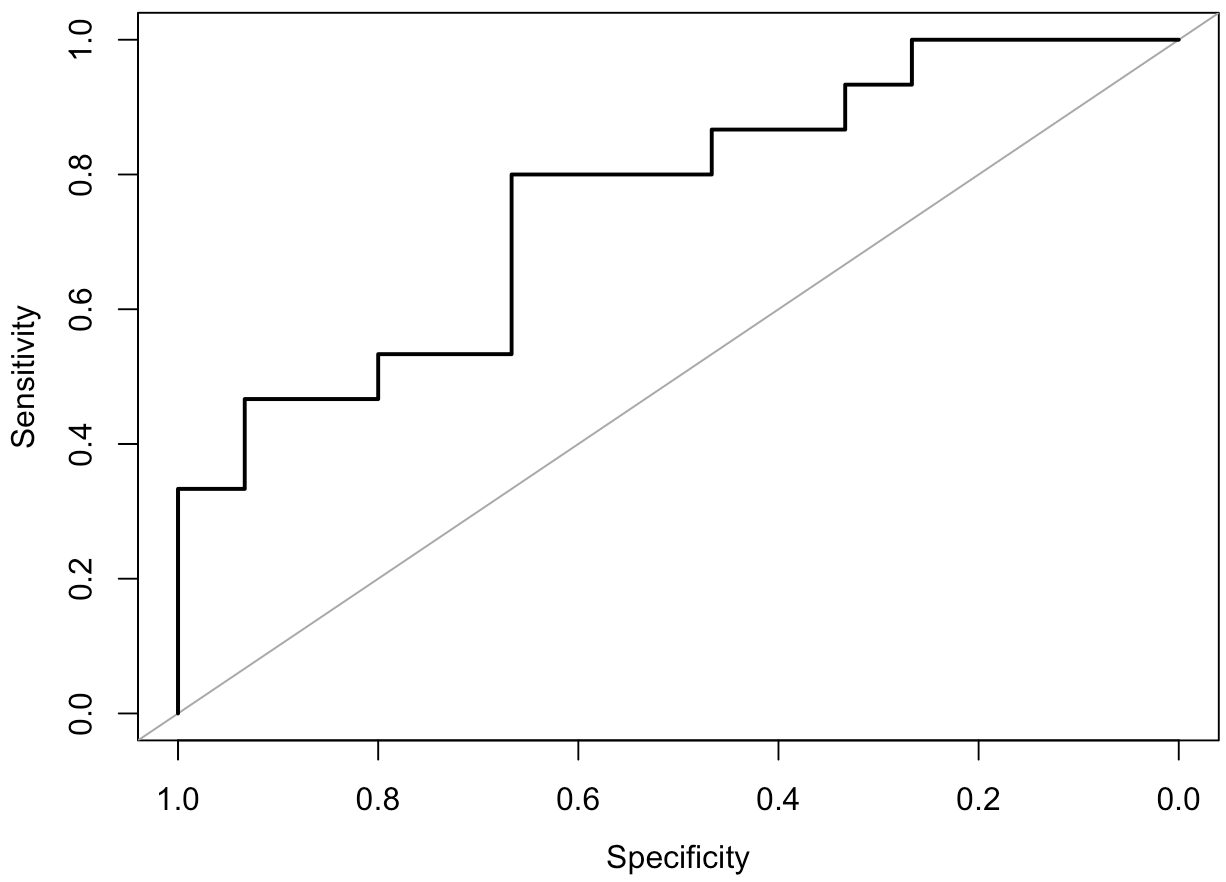


**Supplementary Figure 8 - Receiving Operating Characteristics curve showing the performance of the Random Forest model in classifying ASD vs non-ASD subjects**

The Random Forest model was fed with median DNAm values of the top 50 DMRs identified by performing differential methylation analysis on 70% of the subjects, and then tested for its ability to correctly classify the remaining 30% of the subjects. The overall performance of the model according to the AUC is 0.72.


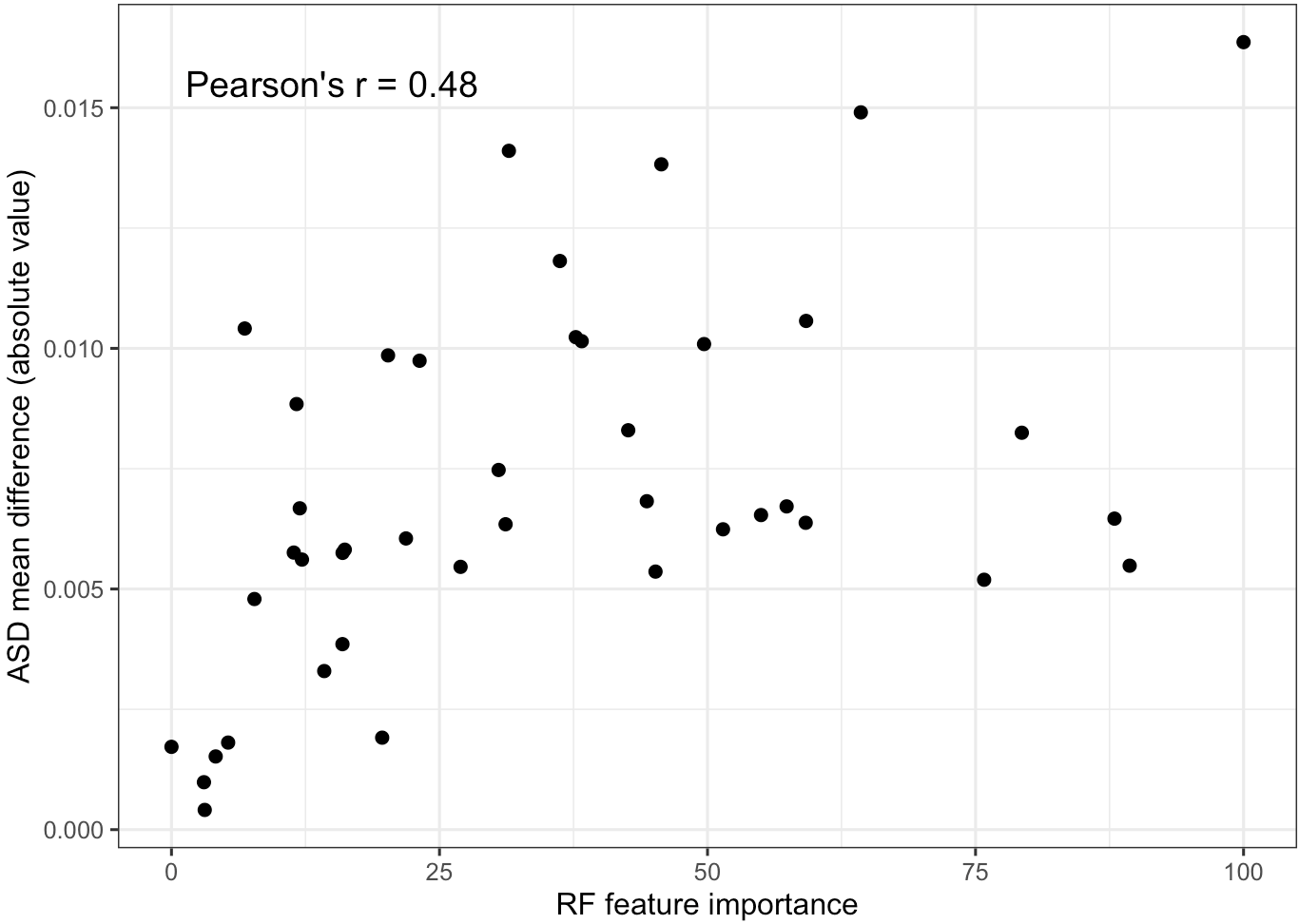


**Supplementary Figure 9 - Correlation between the absolute value of the mean difference (ASD vs control) of the DMRs and their feature importance in the RF model**

The plot shows the correlation between the absolute value of the mean difference (ASD vs control) of the 42 DMRs that overlap between the analysis on conducted on the 70% of the samples and the analysis on the full cohort (on the Y-axis), and their feature importance in the RF-based classification of the test set (Y-axis) shown in Fig 8.


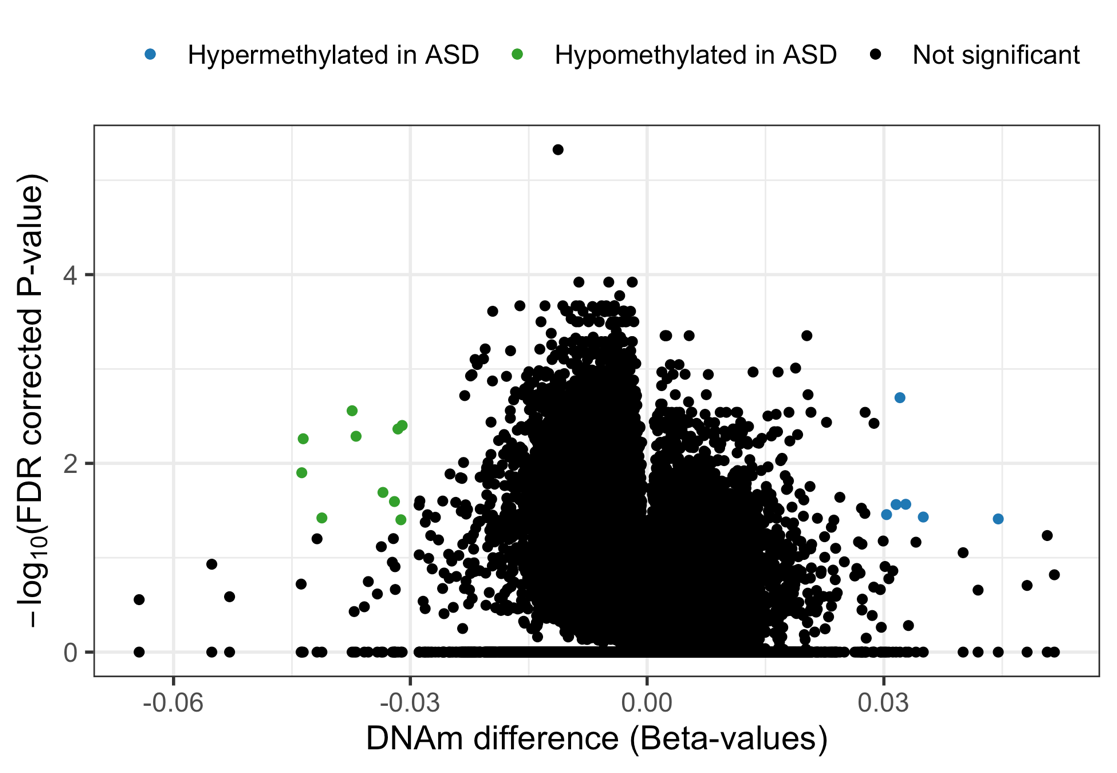


**Supplementary Figure 10 - Volcano plot of distal EPIC probes.** Differential methylation for ASD diagnosis was significant in distal probes with FDR < 0.05 and with a difference in Beta-values between ASD and non-ASD subjects greater (blue points) or smaller (green points) than 0.03.

**Supplementary Table 1-** **Age and number of subjects per ethnicity, diagnosis, and sex.** Age is reported as the average ± standard deviation.

|  |  | **ASD** | | **Non-ASD** | |
| --- | --- | --- | --- | --- | --- |
|  |  | **F** | **M** | **F** | **M** |
| Ethnicity | African | 1 | 6 | 3 | 4 |
|  | Caucasian | 12 | 51 | 24 | 40 |
|  | Hispanic | NA | 1 | NA | 1 |
|  | Other | NA | 4 | 2 | 1 |
| Average Age ± SD |  | 10.3 ± 4.2 | | 11.5 ± 6.2 | |

**Supplementary Table 2** - **Summary of the Dirichlet regression model.** The dependent variables were the cell-type compositions and the independent variable was the diagnosis.

| Cell-type | Intercept $\pm$ SE | ASD diagnosis $\pm$ SE |
| --- | --- | --- |
| B | 2.57 $\pm$ 0.08*** | 0.01 $\pm$ 0.11 |
| CD4T | 3.43 $\pm$ 0.08*** | -0.15 $\pm$ 0.11 |
| CD8T | 2.25 $\pm$ 0.08*** | -0.06 $\pm$ 0.11 |
| Granulocytes | 4.48 $\pm$ 0.07*** | -0.04 $\pm$ 0.11 |
| Monocytes | 2.23 $\pm$ 0.08*** | -0.05 $\pm$ 0.11 |
| NK | 1.70 $\pm$ 0.08*** | -0.96 $\pm$ 0.12*** |

Standard error (SE) followed by *** indicate P-values < 0.001

**Supplementary Table 3 - Contingency table with the number of index DMPs and non-DMPs (at gene level) that are associated with the top ranked neurodevelopmental processes (displayed in blue in Fig. 3), and the number of DMPs and non-DMPs associated with other processed.** The DMPs were overrepresented for neurodevelopment-related processes according to the hypergeometric distribution (one side Fisher’s exact test; odds ratio = 2.96, P-value < 0.001).

|  | DMPs | non-DMPs |
| --- | --- | --- |
| Neurodevelopment | 436 | 138 |
| Other | 12469 | 11670 |

**Supplementary Table 4 -** **Summary of the beta regression model for genetic effects on DNAm of *CLEC11A* and *MOB1A*.** Estimate and standard error (SE) of the intercept describe the mean DNAm of non-ASD subjects on the logit scale. Estimates and SEs of the additional coefficients represent the contribution, relative to the intercept, of ASD and of the two top-ranked mQTL. For each gene, the two genetic variants were in linkage equilibrium and the genotypes were multiplied by the specific Beta-value of the mQTL that was extracted from the GoDMC database.

*CLEC11A MOB1A*

| Coefficient | Estimate $\pm$ SE |  | Coefficient | Estimate $\pm$ SE |
| --- | --- | --- | --- | --- |
| Intercept | -0.80 $\pm$ 0.06*** |  | Intercept | -1.53 $\pm$ 0.03*** |
| ASD | 0.10 $\pm$ 0.05* |  | ASD | -0.05 $\pm$ 0.04 |
| rs4801842 | -0.17 $\pm$ 0.08* |  | rs1020485 | 0.03 $\pm$ 0.06 |
| rs8100750 | 0.20 $\pm$ 0.19 |  | rs4452177 | 0.34 $\pm$ 0.03*** |

Standard errors followed by *** indicate P-values < 0.001, * P-values < 0.05

**Supplementary Table 5 -** Degree of correlation between blood and brain for DMRs associated with TTC23, TBX1, SHANK2 and CLEC11A. The median rho correlation, together with the minimum and maximum rho, for all CpGs included in each DMR is reported, based on data from the Image-CpG database^2^

| DMR | chr | start-end | width | n cpgs | overlapping promoters | median rho (brain-blood) | min rho (brain-blood) | max rho (brain-blood) |
| --- | --- | --- | --- | --- | --- | --- | --- | --- |
| DMR_1 | 15 | 99789622_99790022 | 401 | 8 | TTC23-201, LRRC28-001, LRRC28-002, TTC23-003, TTC23-202, TTC23-001, TTC23-005, TTC23-203, LRRC28-201, LRRC28-202, LRRC28-003, TTC23-002, TTC23-006, TTC23-007, TTC23-016, LRRC28-010, LRRC28-009, LRRC28-020, TTC23-204, TTC23-011, LRRC28-007, LRRC28-008, LRRC28-017, LRRC28-021, LRRC28-005, TTC23-019, TTC23-015, TTC23-017, TTC23-018, LRRC28-022, LRRC28-006, TTC23-014 | 0.28 | 0.16 | 0.61 |
| DMR_2 | 22 | 19749961_19752522 | 2562 | 8 | TBX1-004, TBX1-005 | 0.1 | -0.15 | 0.29 |
| DMR_10 | 11 | 70507825_70508659 | 835 | 9 | SHANK2-202, SHANK2-001, SHANK2-008, SHANK2-016, SHANK2-021, SHANK2-204, SHANK2-205 | 0.1 | -0.17 | 0.39 |
| DMR_26 | 19 | 51225848_51226849 | 1002 | 8 | CLEC11A-001, CLEC11A-003 | 0.13 | -0.17 | 0.17 |

**References**

1 Filosi M, Kam-Thong T, Essioux L, Muglia P, Trabetti E, Spooren W *et al.* Transcriptome signatures from discordant sibling pairs reveal changes in peripheral blood immune cell composition in Autism Spectrum Disorder. *Transl Psychiatry* 2020; 10: 106.

2 Braun PR, Han S, Hing B, Nagahama Y, Gaul LN, Heinzman JT *et al.* Genome-wide DNA methylation comparison between live human brain and peripheral tissues within individuals. *Transl Psychiatry* 2019; 9: 47.
